# Plant virus diversity in bee and pollen samples from apple (*Malus domestica*) and sweet cherry (*Prunus avium*) agroecosystems

**DOI:** 10.3389/fpls.2024.1335281

**Published:** 2024-02-20

**Authors:** Malek Smadi, Eunseo Lee, James Phelan, Aiming Wang, Guillaume J. Bilodeau, Stephen F. Pernal, M. Marta Guarna, Mike Rott, Jonathan S. Griffiths

**Affiliations:** ^1^ London Research and Development Centre, Agriculture and Agri-Food Canada, London, ON, Canada; ^2^ Department of Biology, University of Waterloo, Waterloo, ON, Canada; ^3^ Canadian Food Inspection Agency, Centre for Plant Health, Sidney Laboratory, North Saanich, BC, Canada; ^4^ Canadian Food Inspection Agency, Ottawa Plant Laboratory, Ottawa, ON, Canada; ^5^ Beaverlodge Research Farm, Agriculture and Agri-Food Canada, Beaverlodge, AB, Canada; ^6^ Department of Computer Science, University of Victoria, Victoria, BC, Canada

**Keywords:** plant virus, pollen, metagenomic, apple, cherry

## Abstract

**Introduction:**

Honey bee (*Apis mellifera*) pollination is widely used in tree fruit production systems to improve fruit set and yield. Many plant viruses can be associated with pollen or transmitted through pollination, and can be detected through bee pollination activities. Honey bees visit multiple plants and flowers in one foraging trip, essentially sampling small amounts of pollen from a wide area. Here we report metagenomics-based area-wide monitoring of plant viruses in cherry (*Prunus avium*) and apple (*Malus domestica*) orchards in Creston Valley, British Columbia, Canada, through bee-mediated pollen sampling.

**Methods:**

Plant viruses were identified in total RNA extracted from bee and pollen samples, and compared with profiles from double stranded RNA extracted from leaf and flower tissues. CVA, PDV, PNRSV, and PVF coat protein nucleotide sequences were aligned and compared for phylogenetic analysis.

**Results:**

A wide array of plant viruses were identified in both systems, with cherry virus A (CVA), prune dwarf virus (PDV), prunus necrotic ringspot virus (PNRSV), and prunus virus F (PVF) most commonly detected. Citrus concave gum associated virus and apple stem grooving virus were only identified in samples collected during apple bloom, demonstrating changing viral profiles from the same site over time. Different profiles of viruses were identified in bee and pollen samples compared to leaf and flower samples reflective of pollen transmission affinity of individual viruses. Phylogenetic and pairwise analysis of the coat protein regions of the four most commonly detected viruses showed unique patterns of nucleotide sequence diversity, which could have implications in their evolution and management approaches. Coat protein sequences of CVA and PVF were broadly diverse with multiple distinct phylogroups identified, while PNRSV and PDV were more conserved.

**Conclusion:**

The pollen virome in fruit production systems is incredibly diverse, with CVA, PDV, PNRSV, and PVF widely prevalent in this region. Bee-mediated monitoring in agricultural systems is a powerful approach to study viral diversity and can be used to guide more targeted management approaches.

## Introduction

Apples (*Malus domestica*) and sweet cherries (*Prunus avium*) account for approximately 90% of all tree fruit production in British Columbia (BC), Canada (BC ministry of Agriculture, Food and Fisheries, 2020). Apples and cherries are commonly grown on the same farm to optimize labour costs related to maintenance and harvest due to differences in bloom periods and fruit ripening. Apples and cherries are susceptible to a number of existing and emerging viral pathogens (Umer et al., 2019; Xiao et al., 2022). Due to the longevity of woody tree fruit production systems, individual trees can accumulate multiple viruses over time, negatively affecting production. Apples and cherries both belong to the *Rosaceae* family, and can have similar insect vectors which could facilitate cross-species transmission of pathogens (Umer et al., 2019). Pollination is another major route of plant virus transmission, but little is known regarding the dynamics of interspecies transmission of viruses through pollen (Card et al., 2007; Amari et al., 2009; Fetters and Ashman, 2023).

Rapid apple decline (RAD) has been reported in BC and is a major industry concern. Defined as the sudden and unexplained collapse of usually young (2-8 years) apple trees, RAD is a complex issue and multiple factors have been implicated as a cause including biotic and abiotic factors (Singh et al., 2019; Stokstad, 2019). Viruses have been suggested to be a contributing factor to RAD (Liu et al., 2018), and previous studies of RAD in BC identified 21 viruses and one viroid in declining apple trees in the Okanagan and Similkameen valleys (Xiao et al., 2022). Mixed infections have been reported in BC apples, with up to eight virus species infecting one diseased tree (Xiao et al., 2022). Major viral pathogens of apples include citrus concave gum associated virus (CCGaV; genus *Phlebovirus*), apple mosaic virus (ApMV, genus *Ilarvirus*), apple luteovirus 1 (ALV-1; genus *Luteovirus*), tomato ringspot virus (ToRSV; genus *Nepovirus*), tobacco ringspot virus (TRSV; genus *Nepovirus*), and three major latent viruses from the *Betaflexiviridae* family, apple stem pitting virus (ASPV; genus *Foveavirus*), apple chlorotic leaf spot virus (ACLSV, genus *Trichovirus)*, and apple stem grooving virus (ASGV; genus *Capillovirus*) (Umer et al., 2019; Wright et al., 2020; Xiao et al., 2022).

Cherries are also susceptible to multiple virus species (Umer et al., 2019). Little cherry diseases, caused by little cherry virus 1 (LChV-1; genus *Velarivirus*) and little cherry virus 2 (LChV-2; genus *Ampelovirus*) have been ongoing issues for production in the region since the 1930’s (https://www.bctfpg.ca/pest_guide/info/128/; Reinhold and Pscheidt, 2023). Other viruses can cause substantial losses including cherry virus A (CVA; genus *Capillovirus*), prune dwarf virus (PDV; genus *Ilarvirus*), and prunus necrotic ringspot virus (PNRSV; genus *Ilarvirus*) (Kesanakurti et al., 2017; Simkovich et al., 2021a, b). Prunus virus F (PVF; genus *Fabavirus*), TRSV, ToRSV, and cherry leaf roll virus (CLRV; genus *Nepovirus*). Some of these viruses have been reported to infect both apples and cherries including ApMV, ACLSV, CLRV, ToRSV, TRSV, and PNRSV (Chandel et al., 2008; Hu et al., 2016; Umer et al., 2019; Xiao et al., 2022).

Given the complexity and large number of viruses present in mixed apple and cherry production systems, large scale area-wide monitoring approaches could be beneficial in understanding the diversity of pathogens present, and identifying priorities for management (Roberts et al., 2023). Bee-mediated pollen collection, combined with metagenomic sequencing approaches is a powerful emerging tool in biovigilance and pathogen monitoring (Roberts et al., 2018; Beaver-Kanuya and Harper, 2019; Tremblay et al., 2019; Cunningham et al., 2022; Lee et al., 2023; Tayal et al., 2023). Bee foraging activities essentially sample small amounts of pollen and nectar from multiple flowering individuals within a radius of approximately 2 kilometres from their colony (Steffan-Dewenter and Kuhn, 2003; Couvillon et al., 2014). Many viruses are associated with pollen or bees, and detectable through metagenomics approaches (Card et al., 2007; Pallas et al., 2012; Roberts et al., 2018; Fetters et al., 2022; Fetters and Ashman, 2023; Lee et al., 2023). Here we report the identification of multiple virus species in bee and pollen samples collected from three apple and three cherry sites in Creston valley, BC through bee-mediated metagenomics.

## Methods

### Sample collection

**Figure 1:**
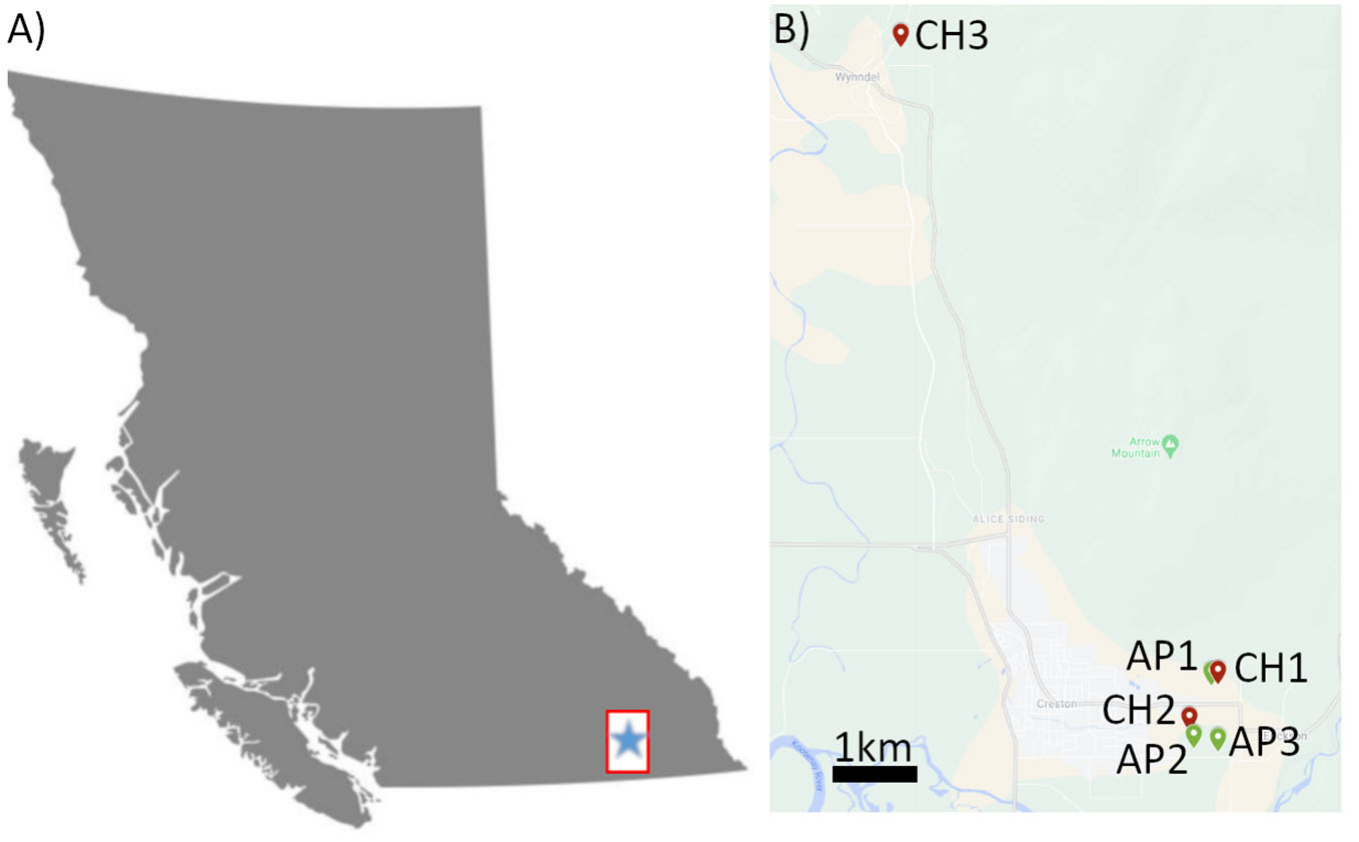
Map diagram of sampling sites in Creston Valley, BC. **(A)** Map of the province of British Columbia. Inset box indicates approximate region of sampling seen in **(B)**. **(B)** Inset map of Creston Valley showing location of sampling sites for Cherry 1-3 (Red icons, CH1-3) and Apples (Green icons, AP1-3). Green points indicate apple sites, red points indicate cherry sites. Scale bar (black) indicates 1 kilometer distance.

Bee and pollen samples were collected from three cherry (CH1-3) and three apple (AP1-3) production sites in the Creston Valley, British Columbia (BC) (**Figure 1**; **Table 1**). Five sites were within approximately a 2 km radius, while cherry site 3 (CH3) was approximately 10 km from all other sites. Multiple bee (*Apis mellifera* L) colonies were located at one site (apiary), and samples were collected from three colonies at each site as biological replicates. Colonies were housed in full-depth Langstroth boxes and were headed by locally-selected queens and a Carniolan strain imported from a commercial queen producer in California. Four different sample types were collected, consisting of a minimum of: 1) 15 individual returning forager bees collected outside the hive with visible pollen in their corbicula; 2) 15 mL of pollen collected from bottom-mounted Ontario agricultural college-style pollen traps, with trays lined with tinfoil (Smith and Adie, 1963); 3) 15 individual hive bees collected from inside the hive without visible pollen on their bodies; and, 4) 5 mL of freshly deposited bee bread collected from one individual frame using new wooden sampling sticks. In addition, eight clusters of blooming flowers and 20 terminal leaf samples were collected from around the circumference of 10 randomly selected apple and cherry trees at each site. These composite leaf/flower samples were divided into two replicate samples prior to sequencing. All samples were placed on dry ice immediately after collection, until being transferred to an ultralow freezer (-80° C) for long-term storage. Cherry samples were collected on May 2^nd^, and apple samples on May 9^th^, 2021 (**Table 1**). Bee colonies were moved out of cherry sites when sampling was completed, and replaced with new colonies at apple sites to reduce potential carry-over of cherry pollen.

**Table 1:** Apple and cherry sampling site details.

Site	Sample names	Crop in bloom during sampling	Varieties	Date sampled	Bee colony GPS location	Plant species on site
1	CHI	Cherry	Staccato, Lapin	May 2nd	49.09808, - 116.4801	Apple, cherry, peach, apricot
	AP1	Apple	Jonagold, Ambrosia, Honeycrisp	May 9^th^	49.09797, - 116.48151	
2	CH2	Cherry	Rainier, Lapin, Sweetheart	May 2nd	49.09179, - 116.48601	Apple, cherry
	AP2	Apple	Jonagold and Spartan	May 9^th^	49.0894, - 116.48504	
3	CH3	Cherry	Lapin	May 2nd	49.18458, - 116.54604	Only cherry
4	AP3	Apple	Gala, Spartan, MacIntosh, Jonagold	May 9^th^	49.08881,- 116.48014	Only apple

### RNA extraction and sequencing

RNA extraction and sequencing was performed as in Lee et al. (2023). Briefly, total RNA (totRNA) was extracted from each bee-related sample using the spectrum total plant RNA extraction kit (Sigma Aldrich, ON, Canada) while double stranded RNA (dsRNA) was extracted from plant samples. dsRNA was extracted from leaf and flower samples following Kesanakurti et al. (2016). Extracted totRNA was treated with a rRNA depletion step using the RiboMinus™ Plant Kit for RNA-Seq (Invitrogen, MA, USA) as per the manufacturer’s instructions. Ribo depleted totRNA and dsRNA HTS libraries were generated using the Illumina TruSeq Stranded mRNA Library Prep kit, following the manufacturer’s protocol, starting after mRNA selection steps (Kesanakurti et al., 2016). An Illumina NextSeq500 was used to generate single-ended 75 base read files to each sample.

### Bioinformatics

HTS sample files were imported into Virtool (www.virtool.ca) for sample management, quality control (QC) and data analysis. Reads passing QC were mapped to known plant virus species in a database updated December 2021 using the Pathoscope 2 pipeline (Kesanakurti et al., 2016). Reads mapping to *P. cerasus, M. domestica*, and *A. mellifera* host genomes were subtracted from further analysis (Hong et al., 2014). Virus identification based on Virtool was used to create sample-specific pathogen profiles. For the purposes of this study, a minimum of 10% genome coverage was required for a virus species to be considered a positive detection from both totRNA and dsRNA samples. Individual sample profiles were combined for each site to create site-specific profiles which included calculating the frequency of detection for each sample type at each site. The average frequency of detection across all samples, average genome coverage, and Viral reads per million (VRPM) for each virus detected across all samples from all sites was also calculated. VRPM is similar to Transcripts Per Million, and was calculated from the total number reads mapping to each individual virus, dividing by genome length of the virus in kilobasepairs (Kbps), and then normalized for the total number of reads in the sequencing run, per million. Due to inconsistencies of apple hammerhead viroid and peach latent mosaic viroid read counts, reads and VRPM, were manually annotated using Geneious prime version 11.0.14.1 (Biomatters inc, CA, USA). RNAseq metadata, calculations for virus detection at cherry and apples sites, and sequence read archive accessions can be found in **Supplementary Files S1**, **S2**, respectively. Statistical significance was calculated using JMP 17.2 statistical software (JMP, NC, USA).

### Pairwise nucleotide sequence analysis and phylogenetics

Using host genome-subtracted *de novo* assembled contigs for each sample, sequences were aligned to the CVA, PDV, PNRSV, and PVF reference sequence using Geneious Prime. Samples with full coat protein (CP) sequence coverage were used for pairwise and phylogenetic analysis. Pairwise nucleotide distance comparisons were constructed using Geneious prime. Maximum likelihood phylogenetic trees were constructed using MEGA 11 with 1000 bootstrap replications (Tamura et al., 2021). CP sequence metadata and genbank accession numbers are detailed in **Supplementary File S3**.

## Results

### Virus detection from managed bee hives in cherry farms

**Figure 2:**
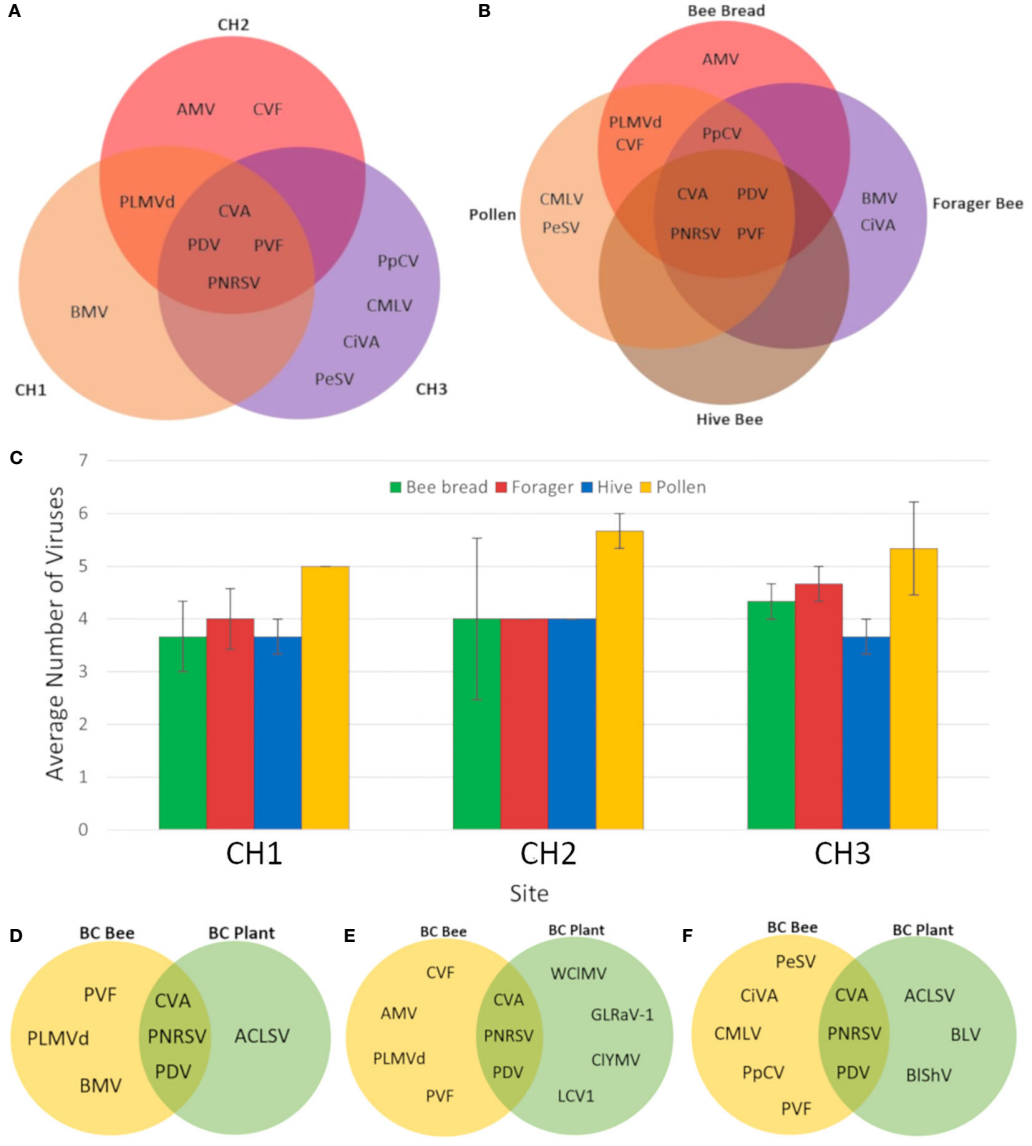
Distribution of viruses identified in different cherry samples and sites. **(A)** Venn diagram of viruses identified in three different BC cherry sites. **(B)** Venn diagram of viruses identified in different sample types from all three BC cherry orchard sites. **(C)** Average number of viruses identified in bee and pollen samples from three BC cherry orchards. Error bars represent standard error. **(D)** Venn diagram of viruses identified in bee and pollen samples compared with plant tissue samples from site CH1. **(E)** Venn diagram of viruses identified in bee and pollen samples compared with plant tissue samples from site CH2. **(F)** Venn diagram of viruses identified in bee and pollen samples compared with plant tissue samples from site CH3. Virus abbreviations: Alfalfa mosaic virus (AMV), apple chlorotic leaf spot virus (ACLV), blueberry latent virus (BLV), blueberry shock virus (BlShV), brome mosaic virus (BMV), cherry mottle leaf virus (CMLV), cherry virus A (CVA), cherry virus F (CVF), citrus virus A (CiVA), clover yellow mosaic virus (ClYMV), little cherry virus 1 (LCV1), grapevine leafroll-associated virus 1 (GLRaV-1), pea streak virus (PeSV), peach latent mosaic viroid (PLMVd), peach mosaic virus (PMV), prune dwarf virus (PDV), prunus necrotic ringspot virus (PNRSV), prunus virus F (PVF), pyrus pyrifolia cryptic virus (PpCV), and white clover mosaic virus (WClMV).

Using a metagenomics-based approach for virus identification in cherry and apple orchards, 12 virus species were identified from bee and pollen samples collected during cherry bloom (**Table 2**). PDV, CVA, PNRSV, and PVF were detected at all three sites, and in all sample types, with frequencies of 100, 97, 97, and 81%, respectively (**Table 2**). Detection frequencies for the other eight viruses were 22% or lower, and often detected at only one site. Four viruses were uniquely detected at site CH3, the most isolated site, including Pea streak virus (PeSV; genus *Capillovirus*), cherry mottle leaf virus (CMLV, genus *Trichovirus*), citrus virus A (CiVA; genus *Coguvirus*) and pyrus pyrifolia cryptic virus (PpCV; genus *Deltapartitivirus*). Peach latent mosaic viroid (PLMVd, genus *Pelamoviroid*) was detected at CH1 and CH2, but was not observed at CH3 (**Figure 2A**). Brome mosaic virus (BMV; genus *Bromovirus*) was only identified at CH1, while cherry virus F (CVF; genus *Fabavirus*) and alfalfa mosaic virus (AMV; genus *Alfamovirus*) were only detected at CH2 (**Figure 2A**; **Table 2**). CVA, PDV, PNRSV, and PVF were identified in all sample types examined (**Figure 2B**). AMV was only detected in bee bread, BMV and CiVA only in forager bees, and CMLV and PeSV only in pollen, while no unique virus species were associated with hive bees (**Figure 2B**). At each site the average number of virus species detected per sample ranged from ~3.5-5.5, no significant differences were observed between sites (two-way ANOVA, F = 0.4714, df = 2, P > F = 0.6282), while the number of viruses identified in pollen was significantly greater than other sample types (two-way ANOVA, F = 4.7226, df = 3, p = 0.0077, not shown). However, when analyzing the number of viruses identified per sample type at each individual site, no significant differences were observed (one-way ANOVA, df = 3, p > 0.05; **Figure 2C**). Viruses identified in totRNA extracted bee and pollen samples were compared to dsRNA leaf and flower samples collected from each site in order to better understand how representative bee-mediated monitoring is of viruses actively replicating in these plants. CVA, PNRSV, and PDV, which were highly prevalent in bee/pollen samples, were also identified in leaf/flower samples, and were the only viruses found to be overlapping between the different sample types. PVF was only detected in bee/pollen samples. A number of viruses, including LCV-1 and ACLSV were only identified in leaf/flower samples (**Figures 2D–F**). Other viruses more common in other host species were also identified including blueberry latent virus, blueberry shock virus, grapevine leaf roll associated virus 1, but had much lower VRPM and average genome coverage compared to CVA, PDV, and PNRSV (**Table 3**).

**Table 2:** Plant virus detections in Bee and Pollen samples from Creston valley cherry sites (CH1, CH2, CH3).

Virus	Genus	Frequency of detection (%)												Average VRPM	Average genome coverage (%)	Average frequency of detection (%)
		CH1				CH2				CH3						
		Bee bread	Forager	Hive	Pollen	Bee bread	Forager	Hive	Pollen	Bee bread	Forager	Hive	Pollen			
		n=3	n=3	n=3	n=3	n=3	n=3	n=3	n=3	n=3	n=3	n=3	n=3			
Prune dwarf virus	llarvirus	100	100	100	100	100	100	100	100	100	100	100	100	105324	84.9	100
Cherry virus A	Capillovirus	100	100	100	100	67	100	100	100	100	100	100	100	5492	87.4	97
Prunus necrotic ringspot virus	llarvirus	100	100	100	100	67	100	100	100	100	100	100	100	21866	78.6	97
Prunus virus F	Fabavirus	33	67	67	100	67	100	100	100	100	67	67	100	346	58.4	81
Peach latent mosaic viroid	Pelamoviroid	33	0	0	100	33	0	0	100	0	0	0	0	29	51.3	22
Pyrus pyrifolia cryptic virus	Deltapartitivirus	0	0	0	0	0	0	0	0	33	67	0	67	19	43.4	14
Cherry virus F	Fabavirus	0	0	0	0	33	0	0	67	0	0	0	0	0	20.4	8
Alfalfa mosaic virus	Alfamovirus	0	0	0	0	33	0	0	0	0	0	0	0	0	11.2	3
Brome mosaic virus	Bromovirus	0	33	0	0	0	0	0	0	0	0	0	0	874	71.9	3
Cherry mottle leaf virus	Trichovirus	0	0	0	0	0	0	0	0	0	0	0	33	0	11.9	3
Citrus virus A	Coguvirus	0	0	0	0	0	0	0	0	0	33	0	0	99	43.8	3
Pea streak virus	Carlavirus	0	0	0	0	0	0	0	0	0	0	0	33	0	10.2	3

**Table 3:** Plant virus detections in leaf and flower samples from Creston valley cherry sites.

Virus	Genus	Frequency of detection (%)			Average VRPM	Average genome coverage (%)	Average frequency of detection (%)
		CH1 n=2	CH2 n=2	CH3 n=2			
Cherry virus A	Capillovirus	100	100	100	2276	95.8	100
Prune dwarf virus	llarvirus	50	100	100	3671	93.9	83
Prunus necrotic ringspot virus	llarvirus	50	100	100	850	54.9	83
Apple chlorotic leaf spot virus	Trichovirus	50	0	50	9	88.1	33
Blueberry latent virus	Amalgaviridae	0	0	50	1	35.3	17
Blueberry shock virus	Bromoviridae	0	0	50	0	10.0	17
Clover yellow mosaic virus	Alphaflexiviridae	0	50	0	0	10.6	17
Little cherry virus 1	Closteroviridae	0	50	0	37	99.7	17
Grapevine leafroll-associated virus	Closteroviridae	0	50	0	0	15.0	17
White clover mosaic virus	Alphaflexiviridae	0	50	0	0	14.3	17

### Virus detection from managed bee hives in apple farms

**Figure 3:**
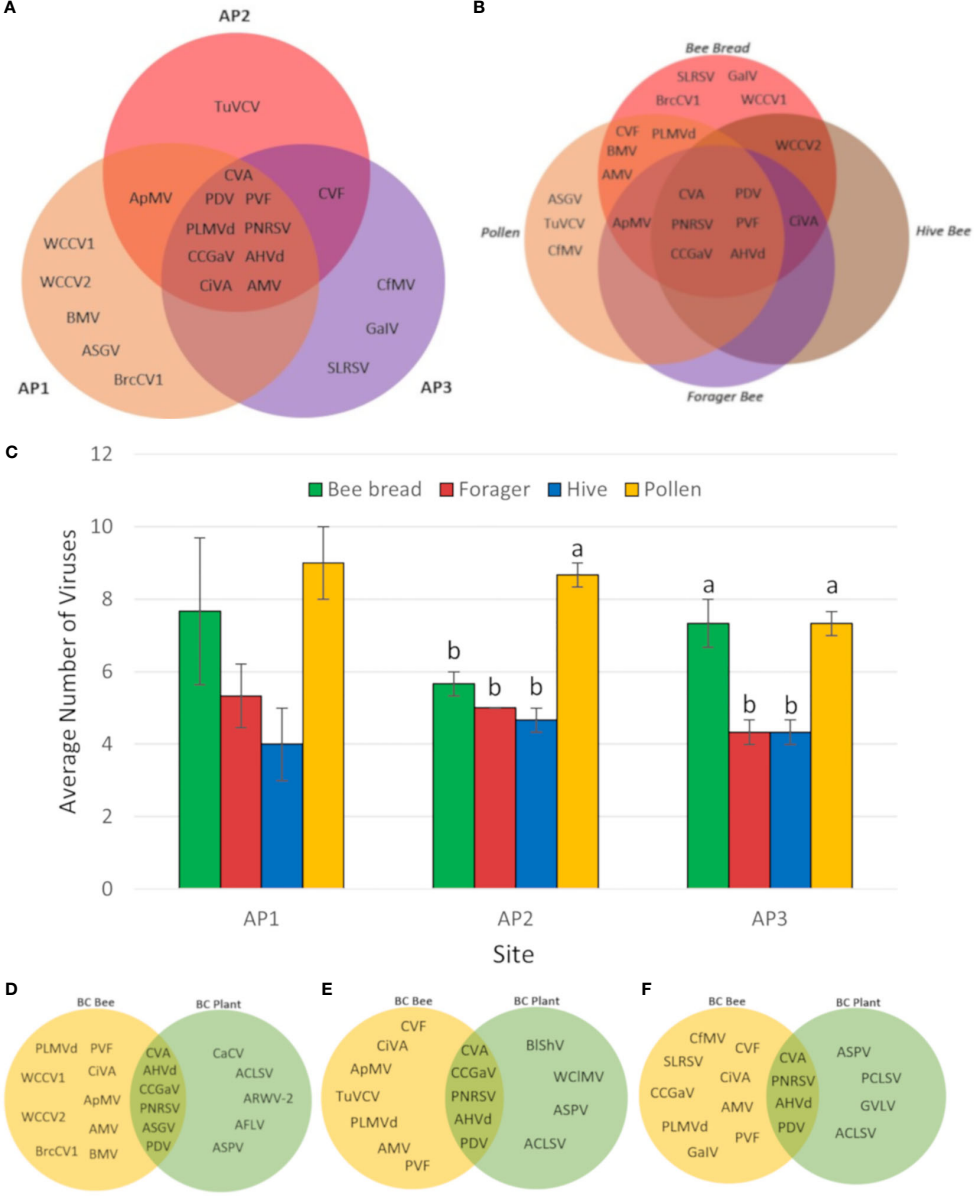
Distribution of viruses identified in different apple samples and sites. **(A)** Venn diagram of viruses identified in three different BC apple orchards. **(B)** Venn diagram of viruses identified in different sample types from three BC apple sites. **(C)** Average number of viruses identified in bee and pollen samples from three BC apple orchards. Error bars represent standard error. Letters indicate significant differences between samples, within locations. (Two-way ANOVA, Tukey’s *post-hoc* analysis, p < 0.05). **(D)** Venn diagram of viruses identified in bee and pollen samples compared with plant tissue samples from site AP1. **(E)** Venn diagram of viruses identified in bee and pollen samples compared with plant tissue samples from site AP2. **(F)** Venn diagram of viruses identified in bee and pollen samples compared with plant tissue samples from site AP3. Virus abbreviations not listed in **Figure 2**: apple hammerhead viroid (AHHVd), apple mosaic virus (ApMV), apple rubbery wood virus 2 (ARWV-2), apple stem grooving virus (ASGV), apple stem pitting virus (ASPV), brassica campestris chrysovirus 1 (BrcCV1), carrot cryptic virus (CaCV), citrus concave gum associated virus (CCGaV), cocksfoot mottle virus (CfMV), grapevine associated ilarvirus (GaIV), grapevine virga-like virus (GVLV), strawverry latent ringspot virus (SLRSV), peach chlorotic spot virus (PCLSV), turnip vein clearing virus (TuVCV), apple flat limb virus (AFLV), white clover cryptic virus 1 (WCCV1), white clover cryptic virus 2 (WCCV2).

A greater diversity of viruses was observed in bee and pollen samples collected during apple bloom, with a total of 20 virus species identified. Similar to cherries, CVA, PDV, PNRSV, and PVF were the most commonly identified across all sample types and sites, with frequencies of 100, 100, 97, and 81%, respectively (**Figure 3A**). Apple hammerhead viroid (AHVd; genus *Pelamoviroid*) was also frequent and, identified in 81% of all samples, while all other viruses had detection frequencies of 31% or lower. CCGaV, citrus virus A (CiVA; genus *Coguvirus*), ApMV, peach latent mosaic viroid (PLMVd; genus *Pelamoviroid*), and AHVd were detected at all three sites (**Figure 3A**). BMV, ASGV, brassica campestris chrysovirus, and white clover cryptic virus 1 and 2 were only identified at AP1, turnip vein clearing virus (genus *Tobamovirus*) only at AP2, and strawberry latent ringspot virus (SLRSV; Secoviridae family), grapevine associated ilarvirus (GalV; genus *Ilarvirus*), cocksfoot mottle virus (genus *Sobemovirus*) at AP3 (**Figure 3A**).

Multiple viruses were identified in all sample types including CVA, PDV, PNRSV, PVF, CCGaV, and AHVd (**Figure 3B**). The average number of viruses identified per sample ranged from 4 to 8.5, and no major differences were observed between sites (two-way ANOVA, F = 0.3164, df = 2, P > F = 0.7310). When examining the frequency of viruses in individual sample types on a per site basis, pollen had significantly greater number of viruses detected at site AP2 (one-way ANOVA, F = 40, df = 3, P > F = 0.0001, Tukey’s HSD, p < 0.05), while both pollen and bee bread samples were significantly greater than forager and hive bee samples (one-way ANOVA, F = 15.4286, df = 3, P > F = 0.0001, Tukey’s HSD, p < 0.05). Viruses unique to bee bread include SLRSV, GalV, WCCV1, BrcCV1, while ASGV, TuVCV, and CfMV were unique to pollen (**Figure 3B**). A dsRNA extraction was also performed on apple leaf and flower tissues to select for viruses actively replicating in these tissues. CVA, PNRSV, PDV, and AHVd were detected in bee/pollen and leaf/flower sample types at all 3 sites, whereas ASGV and CCGaV were only detected in both samples types at AP1. Viruses identified only in plant tissues include ACLSV, apple rubbery wood virus 2 (genus *Rubdobvirus*), AFLV, and ASPV at AP1, BlShV, CCGaV, white clover mosaic virus (genus *Potexvirus*), ASPV, ACLSV from AP2, and ASPV, peach chlorotic leaf spot virus (genus *Trichovirus*), grapevine virga-like virus (unclassified Riboviria), and ACLSV from AP3 (**Figures 3D–F**; **Tables 3**, **4**).

**Table 4:** Plant virus detections in bee and pollen samples from Creston valley apple sites (AP1, AP2, AP3).

Virus	Genus	Frequency of detection (%)												Average VRPM	Average genome coverage (%)	Average frequency of detection (%)
		AP1				AP2				AP3						
		Bee bread	Forager	Hive	Polien	Bee bread	Forager	Hive	Pollen	Bee bread	Forager	Hive	Pollen			
		n=3	n=3	n=3	n=3	n=3	n-3	n=3	n=3	n=3	n=3	n=3	n=3			
Cherry virus A	Capillovirus	100	100	100	100	100	100	100	100	100	100	100	100	325	85.8	100
Prune dwarf virus	Harvirus	100	100	100	100	100	100	100	100	100	100	100	100	2168	86.6	100
Prunus necrotic ringspot virus	llarvirus	100	100	67	100	100	100	100	100	100	100	100	100	576	77.7	97
Apple hammerhead viroid	Pelamoviroid	100	67	33	100	100	100	33	100	100	100	33	100	182	67.7	81
Prunus virus F	Fabavirus	67	67	33	100	100	67	100	100	100	33	100	100	28	64.3	81
Citrus concave gum- associated virus	Coguvirus	33	67	33	100	0	33	0	67	0	0	0	33	121	29.5	31
Citrus virus A	Coguvirus	0	0	0	67	33	0	33	100	33	0	0	33	0	32.3	25
Peach latent mosaic viroid	Pelamoviroid	67	0	0	67	0	0	0	67	33	0	0	33	1	42.8	22
Cherry virus F	Fabavirus	0	0	0	0	33	0	0	33	67	0	0	100	0	34.4	19
Apple mosaic virus	llarvirus	67	33	0	67	0	0	0	33	0	0	0	0	1	18.0	17
Alfalfa mosalc virus	Alfamovirus	0	0	0	33	0	0	0	33	33	0	0	0	0	13.2	8
Brome mosaic virus	Bromovirus	33	0	0	33	0	0	0	0	0	0	0	0	1	32.7	6
White clover cryptic virus 2	Betapartitivirus	33	0	33	0	0	0	0	0	0	0	0	0	4	20.6	6
Apple stem grooving virus	Capillovirus	0	0	0	33	0	0	0	0	0	0	0	0	0	11.1	3
Brassica campestris chrysovirus 1	Alphachrysovirus	33	0	0	0	0	0	0	0	0	0	0	0	0	17.9	3
Cocksfoot mottle virus	Sobemovirus	0	0	0	0	0	0	0	0	0	0	0	33	0	10.9	3
Grapevine associated llarvirus	llarvirus	0	0	0	0	0	0	0	0	33	0	0	0	1	71.2	3
Strawberry latent ringspot virus	unclassified Secoviridae	0	0	0	0	0	0	0	0	33	0	0	0	0	19.7	3
Turnip vein-clearing virus	Tobamovirus	0	0	0	0	0	0	0	33	0	0	0	0	0	13.2	3
White clover cryptic virus 1	Alphapartitivirus	33	0	0	0	0	0	0	0	0	0	0	0	0	10.3	3

### Comparing apples and cherries

**Figure 4:**
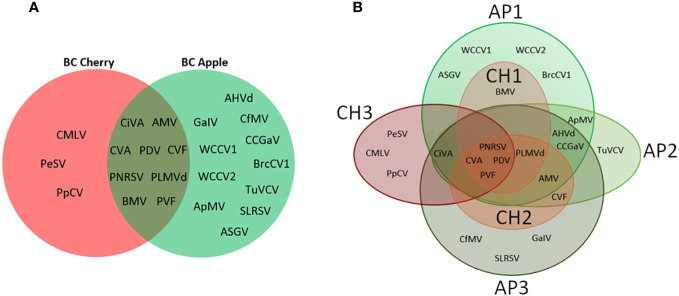
Venn diagram comparisons of viruses detected during cherry or apple bloom. **(A)** Venn diagram of viruses detected at all three cherry sites compared with all three apple sites. **(B)** Venn diagram of viruses identified at individual apple and cherry sites. AP1-3 indicate apple sites 1 through 3, while CH1-3 indicate cherry sites 1 through 3.

Similarities and differences between apple and cherry pollen viromes were evaluated by comparing the diversity of viruses found at each site (**Figure 4A**), and through site-specific comparisons (**Figure 4B**). Nine viruses were common in bee and pollen samples from both sampling time points (CiVA, AMV, CVA, PDV, CVF, PNRSV, PLMVd, BMV, and PVF), but apple samples had a much more diverse virome with 11 unique viruses detected compared to only three in the cherry samples (PeSV, CMLV, PpCV; **Figure 4A**). Although CH1 and AP1 were located on the same grower’s farm (Site 1), sampling times were temporally separated by one week for apple and cherry. Detections of BMV were unique to one site (CH1 and AP1). Similarly, CVF was only identified in CH2 and AP2 at site 2, and the closely located AP3 site.

CVA, PDV, and PNRSV were detected from both apple and cherry samples, and specifically in both bee/pollen samples and leaf/flower samples (**Tables 3**, **5**). CVA and PDV were not expected to be actively replicating in apple trees, since apples are not known hosts for these viruses. Average VRPM and genome coverage from apple leaf/flower samples were much lower compared to cherry samples. For example, in cherries CVA had an average VRPM of 2276 and an average genome coverage of 95.8%, while from apple samples average VRPM was 1 and genome coverage was 47.2% (**Tables 3**, **5**). PDV had an average VRPM of 3671 and an average genome coverage of 93.9% from cherries, while these values were a VRPM of 1 and genome coverage of 36.8 from apples (**Tables 3**, **5**).

**Table 5:** Plant virus detections in leaf and flower samples from Creston valley apple sites (AP1, AP2, AP3).

Virus	Genus	Frequency (%)			Average VRPM	Average Genome coverage (%)	Average frequency of detection (%)
		AP1 n=2	AP2 n=2	AP3 n=2			
Apple chlorotic leaf spot virus	Trichovirus	100	100	100	717	98.8	100
Apple stem pitting virus	Foveavirus	100	100	100	220	92.4	100
Prune dwarf virus	llarvirus	100	100	100	1	36.8	100
Apple hammerhead viroid	Pelamoviroid	100	50	100	2238	100.0	83
Cherry virus A	Capillovirus	100	50	100	1	47.2	83
Prunus necrotic ringspot virus	llarvirus	100	50	100	1	20.1	83
Citrus concave gum associated virus	Coguvirus	100	100	0	76	99.2	67
Apple rubbery wood virus 2	Rubodvirus	50	0	0	5	93.4	17
Apple stem grooving virus	Capillovirus	50	0	0	1785	99.8	17
Blueberry shock virus	llarvirus	0	50	0	0	17.9	17
Carrot cryptic virus	Alphapartitivirus	50	0	0	0	16.3	17
Grapevine virga- like virus	unclassified Riboviria	0	0	50	0	15.2	17
Peach chlorotic leaf spot virus	Trichovirus	0	0	50	0	49.3	17
Apple flat limb virus	unclassified Riboviria	50	0	0	0	97.7	17
White clover mosaic virus	Potexvirus	0	50	0	0	13.6	17

### Viral sequence diversity

CVA, PDV, PNRSV, and PVF were the most frequently detected viruses, identified at all six sites. To characterize the diversity of individual viruses, RNAseq reads were mapped to the coat protein region of all four viruses to create a consensus nucleotide sequence for each sample, which was then used in pairwise analysis (**Supplementary File S3**). 61 CVA CP sequences had identities to each other and the refseq (NC_003689) from 88.5 – 99.7%, 72 PDV CP sequences ranged from 93.2 – 100%, 27 PNRSV CP sequences had identities of 95.3 – 100%, and 20 PVF sequences ranged from 85.5 – 98.2% (**Supplementary File S4**).

**Figure 5:**
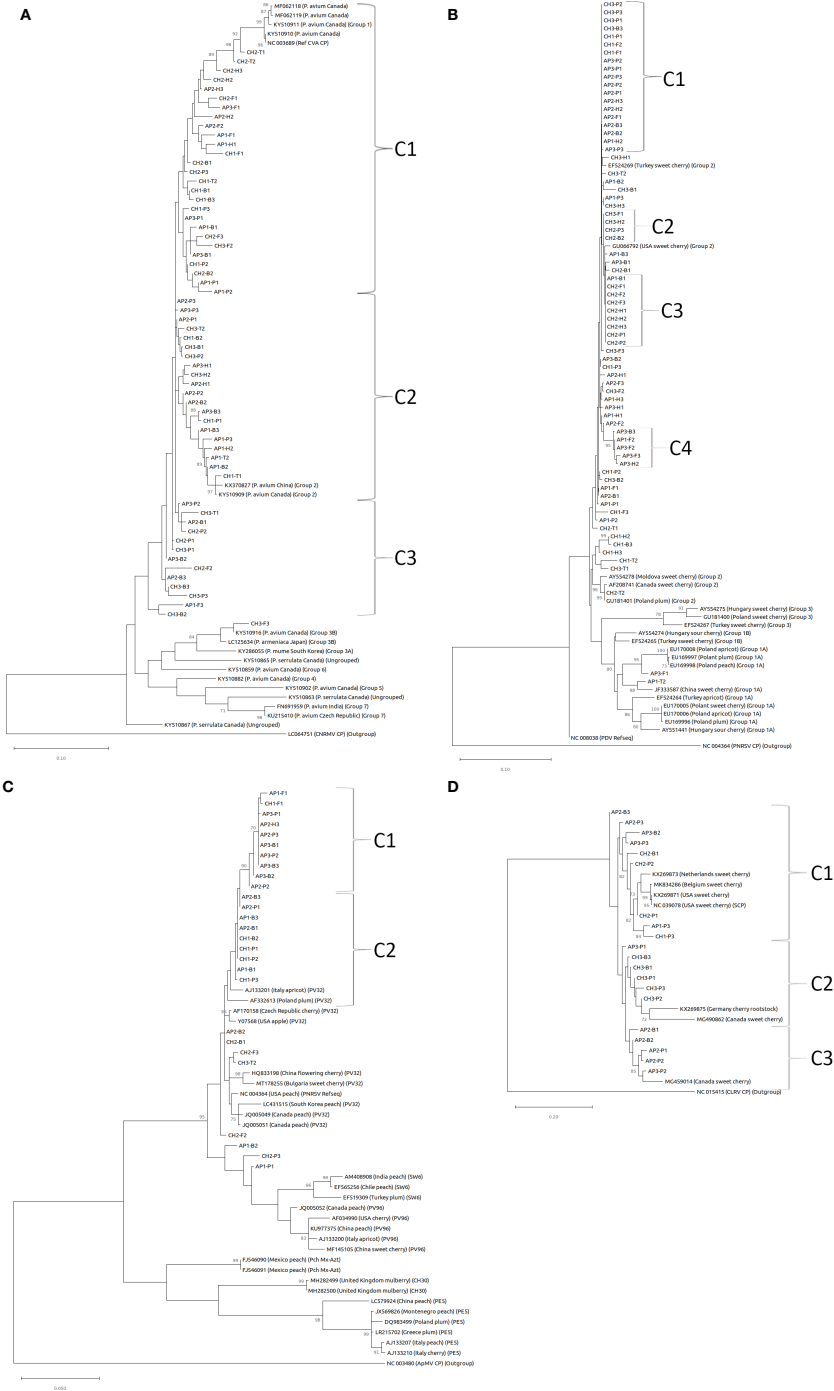
Maximum likelihood phylogenetic tree of coat protein sequences for **(A)** CVA, **(B)** PDV, **(C)** PNRSV, and **(D)** PVF. Bootstrap values (1000 replicates) over 70% are indicated at branch points. Outgroups used in phylogenetic analysis include cherry necrotic rusty mottle virus (CNRMV) in **(A)**, PNRSV in **(B)**, ApMV in **(C)**, and cherry leaf roll virus in **(D)**.

Nucleotide CP consensus sequences for CVA, PDV, PNRSV, and PVF from this study were used to create maximum likelihood phylogenetic trees (**Figure 5**). There are six or seven previously defined major phylogroups for CVA (Gao et al., 2017; Kesanakurti et al., 2017). Sequences from this study were most closely related to phylogroup I and II defined in Gao et al., 2017, and also created new clusters within these groups (**Figure 5A**). 27 sequences map to cluster I (C1), that includes a previously reported Canadian isolate of CVA (MF062118). Two samples from site 2 (CH2-T1 and CH2-T2) recovered from leaf/flower tissue branched more closely with previously reported isolates, while bee and pollen samples were more divergent from these leaf/flower samples within this cluster. One other isolate from leaf/flower tissue (CH1-T2) branched closely with four isolates (CH2-B1, CH2-P3, CH1-B1, CH1-B3) from bee/pollen samples, and were generally >98% identical within this smaller group (**Supplementary File S4**). Another 20 samples in a second major cluster (C2) including two leaf/flower samples (AP1-T2 and CH1-T1), which branched closely with a Canadian isolate from Phylogroup II (KY510909)(Gao et al., 2017). A third cluster (C3) consisting of sequences from 13 samples was also identified. One leaf/flower sample was included in this cluster (CH3-T1), but generally had lower % identity (97.2-97.7%) to other sequences in this cluster. A final isolate (CH3-F3) did not branch with all other sequences from this study, and instead branched more closely with previously reported isolates from Phylogroup 3 (Gao et al., 2017). This isolate generally had low identity (89.8 – 95.6%) to all other isolates from this study (**Supplementary File S4**).

Three major phylogroups have been defined based on CP nucleotide sequence comparisons for PDV (Kinoti et al., 2017). In this study we identified 72 complete PDV CP sequences and most grouped within phylogroup II, including many that formed smaller clusters with 100% identity (**Figure 5B**; **Supplementary File S4**). Most sequences grouped closely with sweet cherry isolates from Turkey (EF524269) and the United States (GU066792)(**Figure 5B**). Four clusters were identified with 99.1 – 100% sequence identity within each cluster. The first cluster (C1) of 19 samples shares 99.8 - 100% identity (**Figure 5B**; **Supplementary File S4**). The second cluster (C2) consisted of nine samples with 100% identity, eight of which were derived from cherry samples. The third cluster (C3) consisting of four cherry samples were also 100% identical within this cluster. Clusters 1-3 all shared 99.7-100% identity between clusters. Finally, the fourth cluster (C4) five apple samples, were 99.1 – 100% identical within this group and were generally more divergent from other sequences in this study (**Figure 5B**; **Supplementary File S4**). One isolate (CH2-T2) branched with other phylogroup II isolates from Canada (AF208741) and Poland (GU181401). Two isolates from apple samples (AP3-F1 and AP1-T2) were the most divergent, with 93-95.9% identity with all other sample isolates, and clustered with sequences from Phylogroup 1 defined in Kinoti et al. (2017) (**Figure 5B**; **Supplementary File S4**).

PNRSV CP nucleotide sequences from this study were highly conserved, and most closely associated with phylogroup PV32 (**Figure 5C**; Aparicio et al., 1999). Two major clusters of sequences (C1 and C2) from this study had especially high sequence identities, ranging from 99.1 – 100% and 99.6 - 100%, respectively (**Figure 5C**; **Supplementary File S4**). Surprisingly, CI consisted primarily (9/10) of apple samples, while CII was a mix of apple and cherry. Three other samples (AP1-B2, CH2-P3, and AP1-P1) were divergent from other sequences identified in this study, and branched more closely with Phylogroups SW6 and PV96 (**Figure 5C**). The lone sequence recovered from leaf/flower sample (CH3-T2) branched closely with one isolate from a forager sample (CH2-F3).

PVF CP nucleotide sequences separated into three clusters (C1, C2, and C3), and were highly variable within and between groupings. The nine sequences in C1 branched closely with isolates from the United States (NC_039078 and KX269871), and shared 90.4 – 96.7% identity within this group (**Figure 5D**; **Supplementary File S4**). CII, consisting of six isolates predominantly from cherry samples (5/6), were 94.7 – 98.2% identical within group, and 87.5 – 96.5% identical to sequences from CI. CII isolates associated with a previously described Canadian isolate from the Okanagan valley (MG490862; James et al., 2018). CIII consisted of five samples obtained during apple bloom, and were 95.9 – 98% identical within group (**Figure 5D**; **Supplementary File S4**). C3 samples clustered closely with another previously described isolate from Canada (MG459014; James et al., 2018).

## Discussion

Metagenomic virus monitoring through bee pollination identified distinct populations of viruses in samples collected during cherry and apple blooms from similar sites in Creston Valley, BC. CVA, PDV, PNRSV, and PVF were widely distributed in both systems, but only PNRSV is known to infect both cherry and apple while the other three viruses are more restricted to *Prunus* host species (Umer et al., 2019). Apples had a more diverse bee/pollen virome compared to cherries, and many viruses were unique to the apple sample collection time point including CCGaV, ApMV, and ASGV. Each of the major viruses detected, CVA, PDV, PNRSV, and PVF had unique patterns of nucleotide sequence variability, which could be used to develop more targeted mitigation approaches. CVA and PVF CP sequences were broadly diverse, and clustered in multiple distinct lineages, while PNRSV and PDV CP sequences were more highly conserved. Some groups with higher internal CP sequence conservation were primarily derived from cherry or apple samples, and could be reflective of changes in host origins. The wide diversity of CP sequences identified in bee and pollen samples was often much greater than that identified through screening of leaf and flower samples. These results further demonstrate the benefits of bee-mediated virus monitoring, and provide novel insights into the expansive virome associated with bee pollination from two major fruit tree crops.

### Bee-mediated virus monitoring and management priorities

Bee-mediated virus monitoring is a powerful and emerging tool that can be used to identify common pathogens, and detect new and emerging threats. PDV, PNRSV and CVA have been reported to be quite common in apple and cherry production systems, which was reflected in bee/pollen based monitoring (Kesanakurti et al., 2017; Umer et al., 2019; Reinhold and Pscheidt, 2023). Bee mediated monitoring identified a few other pathogens that were not detected in leaf/flower tissue including PLMVd, CVF, PVF, and CMLV, demonstrating some advantages to bee-mediated virus monitoring compared to more traditional approaches. Sampling approach, sample sizes and sequencing approaches were not consistent between bee/pollen and leaf/flower samples, but it is important to note that bee-mediated sampling is relatively simple, and can provide a valuable overview of viruses present in the immediate area, and can provide unique insights into viral sequence diversity. PVF in particular was not observed in any leaf/flower samples, but was widely prevalent in bee/pollen samples. Since this virus was not observed in leaf/flower samples, the origins of this virus are unknown, but it is likely to infect cherry trees in the immediate area (Villamore et al., 2016; James et al., 2018). Conversely, LChV-1 and ACLSV, which are not known to be associated with pollen, were only detected in leaf/flower samples. Three viruses were unique to the cherry sampling time points including PpCV, CMLV, and PeSV, of which only CMLV is likely to be infecting cherries. The host range of PpCV includes pear (*Pyrus* spp.), which is more closely related to apple (Osaki et al., 1998; Osaki et al., 2017). CVF was only detected in pollen and bee bread samples from CH2, suggesting this could be an emerging virus at this site and could be a target for further surveys or elimination. Generally, more viruses were identified in pollen samples, suggesting this sample type could be optimum for further monitoring using this approach. Understanding the relationship between host plants, local infection levels, and pollen-based detection methods is key to developing this area-wide monitoring approach, and developing appropriate mitigation strategies.

Cherry and apple samples were collected one week apart, with bee hives replaced in between, which was intended to provide a temporal separation between apple and cherry samples, but despite this some overlap between the bloom periods of the individual tree species did occur, and bees could have foraged from both cherry and apple trees. Comparatively more viruses were identified in apple samples than in cherry samples, which was also reflected in a greater diversity of viruses identified in leaf/flower samples. CVA, PDV, PNRSV, and PVF were the most prevalent viruses detected in both cherry and apple bee/pollen samples, while AHVd, CCGaV, ASGV, and ApMV were unique to apple samples, demonstrating some specificity to the virome of samples collected at similar sites at different time points during fruit tree bloom periods. In addition, CiVA, CCGaV, AHVd, and AMV were identified at each apple site, and CCGaV and AHVd in particular were associated with every sample type. This diversity was mostly due to greater numbers of viruses identified per sample from pollen and bee bread samples.

Viruses identified in bee/pollen samples but absent from leaf/flower samples include CiVA, ApMV, BMV, TuYCV, CCGaV (sites AP2 and AP3), SLRSV, WCCV1, WCCV2, and PLMVd. Many of these viruses (CiVA, ApMV, and CCGaV) are likely infecting apples at these sites, but were not detected through random leaf/flower sampling, while other viruses could be present in cherries nearby (PLMVd), or could be originating from unknown hosts (BMV, TuYCV, and SLRSV). Conversely, viruses identified in leaf/flower tissues not present in bee/pollen samples included CaCV, ACLSV, ARWV-2, AFLV, ASPV, WClMV, GVLV, and PCLSV. Many of these viruses are not known to be pollen associated (ACLSV, ARWV-2, AFLV, ASPV), while others were detected with low confidence (WClMV, GVLV, and PCLSV). Detection of CfMV was unexpected in tree fruit systems, but has previously been reported in BC in orchard grass (*Dactylis glomerata* L.) (Bittman et al., 2006). Many of the viruses identified in bee and pollen monitoring were also identified in a recent survey of viruses associated with diseased and declining apple trees from the nearby Okanagan and Similkameen valleys in BC including CVA, PNRSV, ApMV, ASGV, and Grapevine associated ilarvirus/Solanum nigrum associated ilarvirus (Xiao et al., 2022; Rivarez et al., 2023). In terms of emerging pathogens or identifying priorities for management, CiVA was only detected in bee and pollen samples, which could be resulting from lower infection frequencies. Further surveys could help to eliminate this virus before it becomes more widespread. CVF, CMLV and ASGV were only detected at one individual site, and efforts could be focused in these areas to eliminate emerging viruses before they become more widespread regionally. Cherry site CH3 was the most isolated site (**Figure 4B**), and had the most divergent virome of cherry samples, suggesting site-specific profiles are possible using this approach.

Here we have identified a number of potential threats that can be used to direct mitigation approaches. Identification of low frequency viruses could help to eliminate emerging pathogens before they become established and widespread. Bee mediated plant virus monitoring is a powerful biovigilance tool, that when combined with more targeted surveys, including PCR or ELISA based detection methods, could help to reduce pathogen pressures and improve commercial fruit production (Beaver-Kanuya and Harper, 2019; Cunningham et al., 2022). Viruses with low genetic diversity could be indicative of introductions through clonal propagation methods, while metagenomic based bee monitoring could help to develop strain specific PCR based approaches (Lee et al., 2023).

### Potential for cross species transmission of viruses via bee pollination

Cross-species infections arising from viruses establishing infections outside their normal host range are rare events in plant virology, but can have profound implications. Few experimental studies have examined the potential for cross species transmission of plant viruses through pollen, and few viruses are known to infect both apples and cherries, but virus emergence can be associated with intensive agronomical practices (Elena et al., 2014; Jones, 2018; McLeish et al., 2018; 2019; Fetters and Ashman, 2023). In high density tree fruit production pathogens could be spread through insect or nematode vectors, through pollen, or through mechanical means such as grafting or through contaminated tools. The extent of plant virus transmission between species or even individuals of the same species at an industrial scale are poorly understood. Even within species, cherry and apple varieties have complex pollination requirements and compatibilities, which could have effects on horizontal transmission and viral population structures. CVA, PDV, PNRSV, and PVF were all common in both cherry and apple samples, however only PNRSV has been demonstrated to infect both species. One group of PNRSV sequences were derived primarily from apple samples, which could be reflective of host-specific strains. CVA and PDV read counts and genomic coverage were much lower in apple leaf/flower samples than in cherries (**Tables 2**, **3**). These detections could be due to foreign pollen contamination of the flower samples, and further studies are required to evaluate the unlikely event these viruses are actively replicating in apple tissues.

### Viral nucleotide sequence diversity in pollen

Substantial variation was identified in CP nucleotide sequences recovered from cherry and apple samples, with unique patterns of diversity identified for CVA, PDV, PNRSV, and PVF. Pollen sampling is an effective method of recovering multiple sequence variants from one site, without relying on amplification based approaches to evaluating viral diversity (Oliver et al., 2009; Rott et al., 2017; Kinoti et al., 2018). CVA and PVF sequences were quite diverse (88-100 and 85-99% identity, respectively), and multiple independent lineages were identified at these sites. Conversely, PNRSV and PDV CP sequences were more highly conserved (93-100 and 95-100%, respectively). Further studies of viral pollen diversity could be useful in establishing the extent of virus variation in systems similar to these. Furthermore, viral diversity within one host plant is also poorly understood, and relating virus diversity within one plant to diversity in the pollen produced is another interesting question that could help to establish bottlenecks in viral transmission and evolution.

## Data availability statement

The original contributions presented in the study are included in the article/**Supplementary Files**, further inquiries can be directed to the corresponding author/s.

## Author contributions

MS: Data curation, Formal analysis, Investigation, Visualization, Writing – review & editing. EL: Data curation, Writing – review & editing. JP: Formal analysis, Investigation, Writing – review & editing. AW: Writing – review & editing. GB: Funding acquisition, Project administration, Writing – review & editing. SP: Conceptualization, Funding acquisition, Methodology, Writing – review & editing. MG: Conceptualization, Funding acquisition, Resources, Writing – review & editing. MR: Conceptualization, Data curation, Formal analysis, Funding acquisition, Investigation, Methodology, Software, Supervision, Writing – review & editing. JG: Conceptualization, Data curation, Formal analysis, Funding acquisition, Investigation, Methodology, Project administration, Resources, Supervision, Validation, Visualization, Writing – original draft, Writing – review & editing.

## Supplementary material

Supplementary File S1 Cherry sample virus identification and metadata.

Supplementary File S2 Apple sample virus identification and metadata.

Supplementary File S3 Virus mapped reads and genbank submission data.

Supplementary File S4 CVA, PDV, PNRSV, and PVF CP sequence pairwise identities.

## References

[B1] Amari K. Burgos L. Pallas V. Sanchez-Pina M. A. (2009). Vertical transmission of Prunus necrotic ringspot virus from gametes to seedling. J. Gen. Virol. 90, 1767–1774. doi: 10.1099/vir.0.009647-0 19282434

[B2] Aparicio F. Myrta A. Di terlizzi B. Pallas V. (1999). Molecular variabiity among isolates of prunus necrotic ringspot virus from different prunus spp. Phytopathology 89, 991–999. doi: 10.1094/PHYTO.1999.89.11.991 18944653

[B3] BC ministry of Agriculture, Food and Fisheries (2020) BC Cherry and Apple Acreage report. Available at: https://www2.gov.bc.ca/assets/gov/farming-natural-resources-and-industry/agriculture-and-seafood/animal-and-crops/crop-production/2020_bc_cherry_apple_acreage_report.pdf (Accessed August 10, 2023).

[B4] Beaver-Kanuya E. Harper S. J. (2019). Detection and quantification of four viruses in Prunus pollen: implications for biosecurity. J. virological Methods 271, 113673. doi: 10.1016/j.jviromet.2019.113673 31170470

[B5] Bittman S. Acharya S. N. Hunt D. E. (2006). Chilliwack-VR orchardgrass. Can. J. Plant Sci. 86, 173–175. doi: 10.4141/P05-088

[B6] Card S. D. Pearson M. N. Clover G. R. G. (2007). Plant pathogens transmitted by pollen. Australas. Plant Pathol. 36, 455–461. doi: 10.1071/AP07050

[B7] Chandel V. Rana T. Handa A. Thakur P. D. Hallan V. Zaidi A. A. (2008). Incidence of Prunus necrotic ring spot virus Malus domestica in India. J. Phytopathol. 156, 382–384. doi: 10.1111/j.1439-0434.2007.01361.x

[B8] Couvillon M. J. Schürch R. Ratnieks F. L. W. (2014). Waggle dance distances as integrative indicators of seasonal foraging challenges. PloS One 9, e93495. doi: 10.1371/journal.pone.0093495 24695678 PMC3973573

[B9] Cunningham M. M. Tran L. McKee C. G. Polo R. O. Newman T. Lansing L.. (2022). Honey bees as biomonitors of environmental contaminants, pathogens, and climate change. Ecol. Indic. 134, 108457. doi: 10.1016/j.ecolind.2021.108457

[B10] Elena S. F. Fraile A. Garcia-Arenal F. (2014). Evolution and emergence of plant viruses. Adv. Virus Res. 88, 161–191. doi: 10.1016/B978-0-12-800098-4.00003-9 24373312

[B11] Fetters A. M. Ashman T. L. (2023). The pollen virome: A review of pollen associated viruses and consequences for plants and their interactions with pollinators. Am. J. Bot. 110, e16144. doi: 10.1002/ajb2.16144 36924316

[B12] Fetters A. M. Cantalupo P. G. Wei N. Robles M. T. S. Stanley A. Stephens J. D.. (2022). The pollen virome of wild plants and its association with variation in floral traits and land use. Nat. Commun. 13, 523. doi: 10.1038/s41467-022-28143-9 35082293 PMC8791949

[B13] Gao R. Xu Y. Candresse T. He Z. Li S. Ma Y.. (2017). Further insight into genetic variation and haplotype diversity of cherry virus A from China. PloS One 12, e0186273. doi: 10.1371/journal.pone.0186273 29020049 PMC5636130

[B14] Hong C. Manimaran S. Shen Y. Perez-Rogers J. F. Byrd A. L. Castro-Nallar E.. (2014). PathoScope 2.0: A complete computational framework for strain identification in environmental or clinical sequencing samples. Microbiome 2, 33. doi: 10.1186/2049-2618-2-33 25225611 PMC4164323

[B15] Hu G. J. Dong Y. F. Zhang Z. P. Fan X. D. Ren F. Li Z. N.. (2016). First report of Prunus necrotic ringspot virus infection of apple in China. Plant Dis. 100, 1955. doi: 10.1094/PDIS-01-16-0079-PDN

[B16] James D. Phelan J. Jesperson G. (2018). First report of Prunus virus F infecting sweet cherry (Prunus avium cv. Staccato) in Canada. Plant Dis. 107, 1468. doi: 10.1094/PDIS-12-17-1883-PDN

[B17] Jones R. A. C. (2018). Plant and insect viruses in managed and natural environments: novel and neglected transmission pathways. Adv. Virus Res. 101, 149–187. doi: 10.1016/bs.aivir.2018.02.006 29908589

[B18] Kesanakurti P. Belton M. Saeed H. Rast H. Boyes I. Rott M. (2016). Screening for plant viruses by next generation sequencing using a modified double strand RNA extraction protocol with an internal amplification control. J. Virological Methods 236, 35–40. doi: 10.1016/j.jviromet.2016.07.001 27387642

[B19] Kesanakurti P. Belton M. Saeed H. Rast H. Boyes I. Rott M. (2017). Comparative analysis of cherry virus A genome sequenecs assembled from deep sequencing data. Arch. Virol. 162, 2821–2828. doi: 10.1007/s00705-017-3394-1 28547382

[B20] Kinoti W. M. Constable F. E. Nancarrow N. Plummer K. M. Rodoni B. (2017). Generic amplicon deep sequencing to determine ilarvirus species diversity in Australian Prunus. PloS One 12, e0179284. doi: 10.3389/fmicb.2017.01219 28713347 PMC5491605

[B21] Kinoti W. M. Constable F. E. Nancarrow N. Plummer K. M. Rodoni B. (2018). The incidence and genetic diversity of apple mosaic virus (ApMV) and prune dwarf virus (PDV) in prunus species in Australia. Viruses 10, 136. doi: 10.3390/v10030136 29562672 PMC5869529

[B22] Lee E. Vansia R. Phelan J. Lofano A. Smith A. Wang A.. (2023). Area wide monitoring of plant and honey bee (Apis mellifera) viruses in blueberry (*Vaccinium corymbosum*) agroecosystems facilitated by honey bee pollination. Viruses 15, 1209. doi: 10.3390/v15051209 37243295 PMC10220920

[B23] Liu H. Wu L. Nikolaeva E. Peter K. Liu Z. Mollov D.. (2018). Characterization of a new apple luteovirus identified by high-throughput sequencing. Virol. J. 15, 85. doi: 10.1186/s12985-018-0998-3 29764461 PMC5952423

[B24] McLeish M. J. Fraile A. Farcia-Arsenal F. (2018). Ecological complexity in plant virus host range evolution. Adv. Virus Res. 101, 293–339. doi: 10.1016/bs.aivir.2018.02.009 29908592

[B25] McLeish M. J. Fraile A. Farcia-Arsenal F. (2019). Evolution of plant-virus interactions: host range and virus emergence. Curr. Opin. Virol. 34, 50–55. doi: 10.1016/j.coviro.2018.12.003 30654270

[B26] Oliver J. E. Freer J. Anderson R. L. Cox K. D. Robinson T. L. Fuchs M. (2009). Genetic diversity of Prunus necrotic ringspot virus isolates within a cherry orchard in New York. Plant Dis. 93, 599–606. doi: 10.1094/PDIS-93-6-0599 30764394

[B27] Osaki H. Sasaki A. Nakazono-Nagaoka E. Ota N. Nakune R. (2017). Genome segments encoding capsid protein-like variants of Pyrus pyrifolia cryptic virus. Virus Res. 240, 64–68. doi: 10.1016/j.virusres.2017.07.023 28760347

[B28] Osaki H. Sato Y. Kudo A. (1998). See- and pollen-transmitted double-stranded RNAs detected from Japanese pear. Ann. Phytopathol. Soc Jpn. 64, 110–115. doi: 10.3186/jjphytopath.64.110

[B29] Pallas V. Aparicio F. Herranz M. C. Amari K. Sanchez-Pina M. A. Myrta A.. (2012). Ilarviruses of Prunus spp.: a continued concern for fruit trees. Phytopathology 102, 1108–1120. doi: 10.1094/PHYTO-02-12-0023-RVW 23148725

[B30] Reinhold L. A. Pscheidt J. W. (2023). Diagnostic and historical surveys of sweet cherry (*Prunus avium*) virus and virus-like diseases in Oregon. Plant Dis. 107, 633–643. doi: 10.1094/PDIS-02-21-0327-SR 36018551

[B31] Rivarez M. P. S. Faure C. Svanella-Dumas L. Pecman A. Znidaric M. T. Schonegger D.. (2023). Diversity and pathobiology of an ilarvirus unexpectedly detected in diverse plans and global sequencing data. Phytopathology 113, 1729–1744. doi: 10.1094/PHYTO-12-22-0465-V 37399026

[B32] Roberts J. M. K. Ireland K. B. Tay W. Paini D. (2018). Honey bee-assisted surveillance for early plant virus detection. Ann. App. Biol. 173, 285–293. doi: 10.1111/aab.12461

[B33] Roberts J. M. K. Jooste A. E. C. Pretorius L. S. Geering A. D. W. (2023). Surveillance for avocado sunblotch viroid utilizing the European honey bee (*Apis mellifera*). Phytopathology 113, 559–566. doi: 10.1094/PHYTO-08-22-0295-R 36346373

[B34] Rott M. Xiang Y. Boyes I. Belton M. Saeed H. Kesanakurti P.. (2017). Application of Next generation sequencing for diagnostic testing of tree fruit viruses and Viroids. Plant Dis. 101, 1489–1499. doi: 10.1094/PDIS-03-17-0306-RE 30678581

[B35] Simkovich A. Kohalmi S. E. Wang A. (2021a). First report of little cherry virus 1 infecting sweet cherry in Ontario, Canada. Plant Dis. 105, 3780–4145. doi: 10.1094/PDIS-04-21-0798-PDN 34260282

[B36] Simkovich A. J. Li Y. Kohalmi S. E. Griffiths J. S. Wang A. (2021b). Molecular identification of prune dwarf virus (PDV) infecting sweet cherry in Canada and development of a PDV full-length infectious cDNA clone. Viruses 13, 2025. doi: 10.3390/v13102025 34696454 PMC8541084

[B37] Singh J. Silva K. J. P. Fuchs M. Khan A. (2019). Potential role of weather, soil and plant microbial communities in rapid decline of apple trees. PloS One 14, e0213293. doi: 10.1371/journal.pone.0213293 30840713 PMC6402675

[B38] Smith M. V. Adie A. A. (1963). A new design in pollen traps. Can. Bee J. 74 (4), 4–5, 8.

[B39] Steffan-Dewenter I. Kuhn A. (2003). Honeybee foraging in differentially structured landscapes. Proc. R. Soc B: Biol. Sci. 270, 569–575. doi: 10.1098/rspb.2002.2292 PMC1691282 12769455

[B40] Stokstad E. (2019). Rapid apple decline has researchers stumped. Science 262, 1259. doi: 10.1126/science.363.6433.1259 30898909

[B41] Tamura K. Stecher G. Kumar S. (2021). MEGA11: Molecular evolutionary genetics analysis version 11. Mol. Biol. Evol. 38, 3022–3027. doi: 10.1093/molbev/msab120 33892491 PMC8233496

[B42] Tayal M. Wilson C. Cieniewicz E. (2023). Bees and thrips carry virus-positive pollen in peach orchards in South Carolina, United States. J. Econ Entomol. 116, 1091–1101. doi: 10.1093/jee/toad125 37402628

[B43] Tremblay E. D. Duceppe M. O. Thurston G. B. Gagnon M. C. Cote M. J. Bilodeau G. J. (2019). High-resolution biomonitoring of plant pathogens and plant species using metabarcoding of pollen pellet contents collected from a honey bee hive. Environ. DNA 1, 155–175. doi: 10.1002/edn3.17

[B44] Umer M. Liu J. You H. Xu C. Dong K. Luo N.. (2019). Genomic, morphological and biological traits of the virus infecting major fruit trees. Viruses 11, 515. doi: 10.3390/v11060515 31167478 PMC6631394

[B45] Villamore D. E. Mekuria T. A. Pillai S. S. Eastwell K. C. (2016). High-throughput sequencing identifies novel viruses in nectarines: insights to the etiology of stem-pitting disease. Phytopathology 106, 519–527. doi: 10.1094/PHYTO-07-15-0168-R 26780433

[B46] Wright A. A. Cross A. R. Harper S. J. (2020). A bushel of viruses: identification of seventeen novel putative viruses by RNA-seq in six apple trees. PloS One 15, e0227669. doi: 10.1371/journal.pone.0227669 31929569 PMC6957168

[B47] Xiao H. Hau W. Storoschuk G. MacDonald J. L. Sanfacon H. (2022). Characterizing the virome of apple orchards affected by rapid decline in the Okanagan and Similkameen valleys of British Columbia (Canada). Pathogens 11, 1231. doi: 10.3390/pathogens11111231 36364981 PMC9698585

